# Perityphlitis or Perinephritis

**Published:** 1882-01

**Authors:** Judson B. Andrews


### Perityphlitis or Perinephritis.

Reported by Judson B. Andrews, M. D.

A young man, 21 years of age, in good health, on Monday, Sept. 9, started on horseback for a pleasure excursion in the Adirondack region. During the first three days, a distance of about thirty miles a day was easily made without fatigue or discomfort. On the evening of the fourth day, after a ride of about the same length, a short bath was taken in one of the cool lakes that abound in that locality. It was not followed by chill or even any unpleasant sensation. The journey of the fifth day was begun early in the morning after partaking of a light breakfast of pork and coffee. When twenty miles had been made at about 2 P. M., a hearty meal, prepared by the guide, of pork and potatoes fried in a large quantity of fat, was eaten. A further ride of nine miles, and a supper at the hotel completed the incidents of the day. During the night the patient was taken ill, complaining of a severe pain in the bowels, had a copious movement followed by vomiting. On Saturday morning no breakfast was eaten, the vomiting was repeated, and in the afternoon the bowels moved again freely. The patient, however, continued to suffer from severe pain now located in the right iliac region and kept his bed. He partook of some tea and toast for supper, but without relish, and slept little during the night. On Sunday, while in the recumbent position, the pain was less severe, but the feeling of illness and the pain on motion led to the decision to return home as soon as possible. On Monday morning, he started for the nearest railway station, a distance of thirty miles. This was made in a buckboard wagon. The pain induced by the motion was great and the suffering intense. Some relief was obtained by partially supporting the body by the hands pressed upon the seat of the wagon. From the station, a ride of 120 miles by rail brought the patient to Albany, where the first medical attention was received. Dr. S. B. Ward, Professor of Surgery in the Albany Medical College, was summoned. He found the temperature at 104° and the pulse 120. Under the administration of morphia, rest and relief were obtained. On the morning of the 17th, the temperature reached 104–8°, the pulse still counting 120. The patient, though offered the advantage of hospital treatment, determined to continue the journey home, where he arrived at 1 P. M. The Doctor sent a note in which he stated the result of his examination, as given above, and expressed his fear that the case was one of perityphlitic abscess.

When first seen by myself, soon after reaching home, the temperature was then 105, the pulse 130, and the pain in the right groin was so severe that the leg could not be moved voluntarily. The pain was reflected along the cord and felt severely in the testicle. There was retention of urine; the catheter was employed and its use continued twice a day till the following Saturday. Hot fomentations were applied. An ounce of whisky was given every two hours. A single dose of 20 grs. of quinia, and morphia ⅛ gr., the latter to be repeated till pain was relieved, were administered. Milk was taken freely and at regular intervals. In the morning, the temperature stood 103° and the pulse at 112. The pain in the groin had mostly disappeared, and the leg could be moved voluntarily. From this time forward for several days, the temperature varied from 102¼ to 104, and the pulse fell to 96.

On Wednesday, the 18th, an attack of vomiting occurred, but ceased on the stomach being relieved of its contents. The following day the bowels were tympanitic, and by pressure upon the diaphragm produced nausea. This was relieved, and a good movement obtained by an enema.

During the week the pain was not severe, but was felt on pressure along the lower border of the ribs, and especially over the right kidney. The same line of treatment was continued; whisky to the amount of 24 ounces, quinia from 20 to 40 grs. and morphia from ½ to 1 gr. daily. The patient had no chill, sweating, vomiting or delerious. There was no dryness of the tongue, nor sordes upon the teeth, and considering the gravity of the disease, was in a very comfortable condition. Examination of the urine discloses the presence of a slight amount of albumen, about three-tenths of one part in a thousand.

On Monday the 23d inst., there was some thickening of the abdominal parietes, in the right iliac fossa, and on Tuesday a swelling was detected. The temperature increased to 105°, the pulse to 120 and the pain was severe. The necessity for operative interference became apparent, and Dr. Ward was called in consultation. Examination on Tuesday evening and again on Wednesday morning, failed to give positive indications of the presence of pus, and the operation was delayed. On the evening of Wednesday, swelling and redness of tissues were apparent above Poupart's ligament and near the external opening of the inguinal canal. The tissues were still hard and tense, but deep fluctuation was thought to be detected. On Thursday morning, the diagnosis was further confirmed, and an operation decided upon. This was performed by Dr. Ward, assisted by Drs. Ford, Brush and Andrews. The temperature was 105, and the pulse 140. Ether was administered, and an exploration by an hypodermic needle inserted perpendicularly to the surface revealed the presence of pus. An incision was made an inch and one-half in length just above the ligament, and about an inch inside of the course of the external iliac artery. Pus flowed freely to the amount of one pint; it had the characteristic fæcal odor and contained flakes of lymph. The patient bore the anæsthetic and the operation well, and in the afternoon the temperature fell to 102½ and the pulse to 120.

The temperature continued to vary for 102½ to 104, the discharge being free and the pus normal till Saturday evening, the 28th of September, when an abscess was developed under Poupart's ligament, directly over the femoral vessels. This was opened, and a quantity of sanious pus discharged. There was a communication between the two openings, and the carbolized water used for washing out the upper abscess flowed freely from the lower one, till both were closed by the healing process. The next night the temperature again reached 105; the patient was restless and slept poorly. The following morning it stood at 104, the pus was sanious, ill-conditioned and scanty, and on examination showed the presence of uric acid. In the evening the temperature was 105; the patient was dull and sleepy, perspiration profuse, and the feebleness very marked. Poultices were kept applied to the wounds, the whiskey was increased to two ounces every hour, and quinia given in a 15-gr. dose, repeated at midnight. At 6 A. M., of Tuesday, the temperature stood at 101½. The morning urine contained blood and pus. This increased the fear induced by the discovery of the preceding day, that there might be some connection between the abscess and the urinary tract. To reduce the interchange of material to a minimum, it was determined to keep the bladder empty by leaving a catheter in it. This produced so much discomfort that after a few hours it was given up and frequent urination insisted upon. In the afternoon the pus had improved in character and amount, the patient was taking food more readily, and was feeling much better; temperature 103°.

Wednesday evening, the opening of the lower wound was enlarged.

On Friday morning, October 4, the temperature stood at 102°, and thorough examination of both urine and pus showed there was no admixture; it increased to 104½ in the evening, and the symptoms became more severe. The cause of the unfavorable change was not apparent and created considerable anxiety, only added to when at 2 A. M. of Saturday all discharge from the wound ceased. This had been thoroughly washed out every two or three hours. At the washing at 6 A. M. a small shred floating from the upper opening was found attached to a mass of disorganized material, so large that it could be extracted only when compressed with the dressing forceps. There followed immediately about six ounces of decomposed pus. This came from the region of the bladder near the root of the penis. The patient had complained for some days of pain and tenderness in this region. The temperature commenced to fall, and at 9 A. M. reached 101½, but stood again in the evening at 104°. There was for a few days no special change in the condition of the patient, who was improving in appearance, strength and appetite; tongue was clean and wound healing, the discharge decreasing in amount. On the 8th of October he passed a large lumbricoid, 10 inches long. October 10, examination of urine made by Mr. Deecke, special pathologist of the Utica Asylum, with the following result: deposit of urate of soda, urate of ammonia, phosphates and pus corpuscles; temperature again elevated, reaching 105. Quinia and whiskey increased during the night, and morning temperature 102.

Oct. 13. Urine again examined; result, sp. g. 1023, reaction acid, deposit, pus corpuscles, transparent casts, a few granular casts, epithelium from pelvis of kidney and bladder mucus, traces of albumen. From this time stimulants were reduced, and lactic acid in 10 gtt. doses given.

Oct. 18. Urine shows little evidence of cystitis, and no albumen. Microscopically, *many casts and much epithelium from the lining of the uriniferous tubes.*

Oct. 21. Reaction acid; no albumen; microscopically same as before. Patient is still gaining in strength, eating more freely, tongue clean, bowels moving regularly, as they have done during most of the sickness. Discharge from wounds diminishing, and they are filling up from the bottom.

Oct. 24. There is a bagging of pus from the upper wound, toward the iliac fossa, and a free incision, with patient under ether, discloses a secreting membrane; wound kept open by pledget of lint; stimulants are reduced to sherry wine, and tonic doses of quinia are only given. Iodide of potass given in 20-gr. doses b-d, with special reference to kidney trouble. Amount of urine has not fallen below three pints per day, and there is no pain in region of kidney or difficulty of urination. For a few days there has been some sciatic neuralgia in left limb, and slight œdema about ankles. The pulse persists in its rapidity of beats from 112 to 120.

Oct. 27. Examination of urine shows presence of casts. Patient is now able to be moved from bed to lounge; discharge from wound small and decreasing; improvement in general condition marked; appetite good; takes ale only as a tonic. Quinia discontinued.

Oct. 30. On crutches and able to be carried down stairs.

Nov. 1. Took a short ride in carriage.

Nov. 2. All discharge has ceased; external wounds closed; temperature 99; pulse 120. Urine same as last examination, *transparent casts.*

Nov. 6. Patient took ride again; is feeling well and steadily gaining.

Nov. 10. Temperature varies from 99 to 100°; pulse from 112 to 120; slight œdema about ankles; casts still present, but decreasing in amount. Iodide potass continued. Patient is considered convalescent.

In considering the case I cannot avoid the conclusion that we have been dealing with a perinephritis and not a perityphlitis; that there has been an inflammatory condition essentially chronic in character, and which I fear will result in partial cyrrhosis of one or a part of one of the kidneys. It may not be of so serious a character, and after a season may entirely subside without any apparent unpleasant results.

The case has been characterized by an extremely high temperature, and since the operation a very rapid pulse. There have been a series of incidents or accidents which have called for the strictest watchfulness.

